# Arginine is neuroprotective through suppressing HIF-1α/LDHA-mediated inflammatory response after cerebral ischemia/reperfusion injury

**DOI:** 10.1186/s13041-020-00601-9

**Published:** 2020-04-22

**Authors:** Song-Feng Chen, Meng-Xian Pan, Jun-Chun Tang, Jing Cheng, Dan Zhao, Ya Zhang, Hua-Bao Liao, Rui Liu, Yang Zhuang, Zhi-Feng Zhang, Juan Chen, Rui-Xue Lei, Shi-Fang Li, Huan-Ting Li, Ze-Fen Wang, Qi Wan

**Affiliations:** 1 Department of Physiology, School of Basic Medical Sciences, Wuhan University School of Medicine, 185 Donghu Street, Wuhan, 430071 China; 2 Department of Neurosurgery, Renmin Hospital of Wuhan University, 99 Zhang Zhidong Rd, Wuhan, 430060 China; 3 Department of Physiology, School of Basic Medical Sciences, Hubei University of Medicine, 30 South Renmin Road, Shiyan, 442000 Hubei China; 4 Department of Neurology, the Central Hospital of Wuhan, Tongji Medical College, Huazhong University of Science & Technology, 26 Shengli Street, Wuhan, 430013 China; 5 Department of Neurosurgery & Pathophysiology, Institute of Neuroregeneration & Neurorehabilitation, Qingdao University, 308 Ningxia Street, Qingdao, 266071 China

**Keywords:** Arginine, Neuroinflammation, Ischemia stroke, LDHA, HIF-1α, Neuroprotection

## Abstract

Neuroinflammation is a secondary response following ischemia stroke. Arginine is a non-essential amino acid that has been shown to inhibit acute inflammatory reaction. In this study we show that arginine treatment decreases neuronal death after rat cerebral ischemia/reperfusion (I/R) injury and improves functional recovery of stroke animals. We also show that arginine suppresses inflammatory response in the ischemic brain tissue and in the cultured microglia after OGD insult. We further provide evidence that the levels of HIF-1α and LDHA are increased after rat I/R injury and that arginine treatment prevents the elevation of HIF-1α and LDHA after I/R injury. Arginine inhibits inflammatory response through suppression of HIF-1α and LDHA in the rat ischemic brain tissue and in the cultured microglia following OGD insult, and protects against ischemic neuron death after rat I/R injury by attenuating HIF-1α/LDHA-mediated inflammatory response. Together, these results indicate a possibility that arginine-induced neuroprotective effect may be through the suppression of HIF-1α/LDHA-mediated inflammatory response in microglia after cerebral ischemia injury.

## Introduction

Stroke occurrence leads to high death rate and serious disability. Ischemia stroke accounts for 85% of different types of stroke [1]. After ischemic phase, the following reperfusion causes the secondary stage of injury, involving neuroinflammation, oxidative stress and blood-brain-barrier break [2, 3]. The extension of post-ischemia inflammation is related to poor clinical outcome. Harmful molecules are released after ischemia phase and result in brain sterile inflammation with the presence of parenchyma infiltration and cytokine release. Due to the complexity, the mechanism of neuroinflammation is not clearly demonstrated yet [4, 5].

The inflammation process is firstly mediated by microglia [5]. Once microglia are activated to be pro-inflammatory, the neutrophils are recruited. Subsequently, neutrophils, monocytes and lymphocytes are further attracted, forming a vicious cycle [5, 6]. Microglia makes up 5–20% of the glia cells and 10% of brain cells. There are two types of activated microglia that are pro-inflammatory and anti-inflammatory. Pro-inflammation microglia synthesis and release pro-inflammatory molecules such as iNOS, CD32, IL-1β; The anti-inflammation microglia synthesis and release anti-inflammatory molecules such as YM-1, CD206 and IL10. Pro-inflammation is detrimental and does further damage to the ischemia tissue, while the anti-inflammation is neuroprotective and promotes functional recovery [7–9]. Thus, attenuating pro-inflammation and enhancing anti-inflammation in microglia would avoid severe inflammatory response and thereby protect against neuronal death after cerebral ischemia-reperfusion (I/R) injury.

Arginine is a non-essential amino acid. It can be metabolized by Arg (arginase), NOS (nitric oxide synthase), ADC (arginine decarboxylase) and AGAT (arginine-glycine amidinotransferase) [10, 11]. It is reported that arginine has protective effect in a mouse model hind limb ischemia-reperfusion injury. Arginine enhances young rabbit growth and immunity by regulating intestinal microbial community [12, 13]. Arginine is essential for cell survival and the deprivation of arginine leads to apoptosis [14]. Changes in L-arginine metabolism by Sema4D deficiency induce promotion of microglial proliferation in ischemic cortex [15]. The role of arginine in inflammatory response in cerebral I/R injury is not investigated.

HIF-1α (hypoxia inducible factor 1α) increases in response to ischemia stroke and HIF-1α inhibition attenuates inflammatory response [16, 17]. Inhibiting HIF-1α is reported to suppress M1 microphage, iNOS and COX2 pro-inflammatory activity [18]. In addition, HIF-1α interacts with glycolysis-related genes such as PKM2 [19]. LDHA (lactate dehydrogenase) is downstream of HIF-1α and is reported to promote inflammation in multiple sclerosis [20]. However, the LDHA-mediated inflammatory response is not investigated in detail. In spite that LDHA is the downstream effector of HIF-1α and mediates the inflammatory response, the role of LDHA in ischemia stroke remains largely unknown.

In this study, we show that arginine exerts neuroprotective effect in cerebral ischemia injury by suppressing microglia-mediated inflammatory response. Our data indicate that arginine confers neuroprotection through suppressing HIF-1α/LDHA signaling pathway in microglia, which inhibits the inflammatory response after cerebral I/R injury.

## Method

### Animals

Adult male Sprague-Dawley (SD) rats of 250 to 300 g weighing, were group-housed with 2–3 rats per cage on a 12 h light/dark cycle in a temperature-controlled room (23 °C–25 °C), with free access to food and water. Animals were allowed at least 3 days to acclimatize before experimentation. The total number of male rats used in our in vivo experiments was 210. Seventeen adult pregnant female rats and seventy embryos were used for primary cortical neuronal and microglia cultures. All animal use and experimental protocols were approved and carried out in compliance with the IACUC guidelines and the Animal Care and Ethics Committee of Wuhan University School of Medicine. Samples were assigned to the experimental groups by randomize, for data collection and procession. The experiments were performed by investigators blinded to the groups for which each animal was assigned. All studies involving animals are reported in accordance with the ARRIVE guidelines for reporting experiments involving animals.

### Focal cerebral ischemia

Transient focal cerebral ischemia was operated by suture occlusion technique. The whole process is applied as our laboratory described [21]. Male SD rats weighing 250–300 g were anesthetized with 4% isoflurane in 70% N_2_O and 30% O_2_ using a mask and body temperature was remained during and after surgery. The middle incision was made on the neck, the right external carotid artery (ECA) was carefully exposed and dissected. A 3–0 monofilament nylon suture was inserted from the ECA into the right internal carotid artery to occlude the origin of the right middle cerebral artery (MCA) (approximately 22 mm). After 90 min of occlusion, the suture was removed to allow reperfusion, the ECA was ligated, and the wound was closed. Sham-operated rats underwent identical surgery and/or intracerebroventricular injections except that the suture was inserted and withdrawn immediately. Behavioral tests were applied before and after middle cerebral artery occlusion (MCAO) at day − 1, 1, 3, 7 and 14. Brain samples were acquired after 24 h after MCAO, before reperfusion with ice-cold 0.9% saline and decapitated, rats were anesthetized with 4% isoflurane in 70% N_2_O and 30% O_2_. The peri-infarct tissues of ipsilateral hemispheres of the brains were rapidly removed for Western blot test, RT-PCR and the sliced brain for TTC (2,3,5 -triphenyltetrazolium chloride) staining. At 1 h after MCAO insult, arginine (1.0 mg/kg) (A5006, Sigma, USA) was administrated.

### Infarct measurement

The brain was placed in a cooled matrix, and was cut into 2 mm coronal sections. Individual sections were placed in 10-cm petri dishes and soaked in a solution of 2% TTC in PBS at 37 °C and incubated for 30 min. The slices were fixed in 4% paraformaldehyde at 4 °C for 24 h. All image collection, processing, and analysis were performed in a blinded manner and under controlled environmental lighting. The scanned images were analyzed using image analysis software (Image-Pro Plus version 6.0) [22].

### I.c.v. injection administration

Rats were anesthetized with a mixture of 4% isoflurane in 30% O_2_ and 70% N_2_O in a sealed perspective box. Once rats were deeply anesthetized, the ear bars and an upper incisor bar fixed the rat’s head in a stereotaxic frame, and the rats were anesthetized continuously with 4% isoflurane by a mask. A small sagittal incision was made with bregma as the anatomical reference point. A 23-gauge needle attached to a Hamilton microsyringe via polyethylene tubing and infused the drug at a rate of 1.0 μl/min to the cerebral ventricle (from the bregma: lateral, 1.5 mm; anteroposterior, 20.8 mm; depth, 3.5 mm). Proper needle placement was verified by withdrawing a few microliters of clear cerebrospinal fluid into the Hamilton microsyringe [22].

### Cell culture

BV-2 cells were purchased from the Chinese Academy of Sciences Cell Bank. Cells were seeded in a six-well plate (approximately 8 × 105 cells per well) in DMEM supplemented with 10% heat-inactivated FBS, penicillin G (100 U/ml), streptomycin (100 mg/ml), and L-glutamine (2.0 mM) and incubated at 37 °C in a humidified atmosphere containing 5% CO_2_ and 95% air. When the confluence of BV-2 cells reached 60–70% on the treatment day, the cells were transfected with mouse HIF-1α WT plasmid or LDHA WT plasmid by lipo2000 Lipofectamine 2000 (Invitrogen, USA) following the manufacturer’s instructions. The medium was then replaced with normal growth medium for 24 h. On the following day, the cells were then prepared for treatment or Western blot analysis.

### Cortical neuron culture

Cortical neuron cultures were prepared from female SD rats at 17 d of gestation. The pregnant rats were anesthetized then killed by cervical dislocation. The rats were sprayed with 70% ethanol and embryos were removed then quickly decapitated. The cortices were placed in ice-cold plating medium (neurobasal medium (21,103,049, Gibco, USA), 0.5% FBS (16,140,071, Gibco, USA), 2% B-27 supplement (17,504,044, Gibco, USA), 25 mM glutamic acid (IG0710, Solarbio, China), and 0.5 mM L-GlutaMAX (35,050,061, Gibco, USA)) following the removal of the meninges. The cortical neurons were plated on petri dishes coated with poly-D-lysine (P6407, Sigma, USA) and suspended in plating medium. Half of the plating medium was removed and replaced with maintenance medium (neurobasal medium, 0.5 mM L-GlutaMAX, and 2% B-27 supplement) in the same manner every 3 d. The cultured neurons were used in the experiments after 12 d.

### Cortical microglia culture

Cortical microglia cultures were prepared from female SD rats at 17 d of gestation. The pregnant rats were anesthetized then killed by cervical dislocation. The rats were sprayed with 70% ethanol and embryos were removed then quickly decapitated. The brain tissue of embryos was quickly separated. Pelleted cells were resuspended in warmed DMEM culture medium completed with 10% heat inactivated FBS, 1% antibiotic-antimycotic, and 5 ng/ml carrier-free recombinant mouse GM-CS. The cortical microglia were plated into tissue culture–grade poly L-lysine–coated T75 cell culture–treated flasks and placed in a 37 °C incubator with relative humidity. The culture supernatant was replaced twice weekly with 10 ml of fresh completed medium until confluency of cells was observed at approximately 3 weeks.

### Oxygen-glucose deprivation insult

Cells were transferred to a glucose-free extracellular solution (116 mM NaCl, 0.8 mM MgSO_4_, 5.4 mM KCl, 1.0 mM NaH_2_PO_4_, 26 mM NaHCO_3_, and 1.8 mM CaCl_2_) and deoxygenated environment, placed in a humidified chamber (Plas-Labs, Lansing, MI), and maintained at 37 °C in 5% H_2_/85% CO_2_/10% N_2_ for 60 min. Next, the cells were replaced with fresh maintenance medium containing an appropriate concentration of reagents for 24 h in a 5% CO_2_/95% O_2_ incubator. The control cultures were first transferred to another extracellular solution (5.4 mM KCl, 116 mM NaCl, 0.8 mM MgSO_4_, 1.8 mM CaCl_2_, 1.0 mM NaH_2_PO_4_, 26 mM NaHCO_3_, and 33 mM glucose) and placed in the humidified chamber, which was maintained at 37 °C in 95% O_2_/5% CO_2_ for 60 min. Finally, replaced with fresh maintenance medium for the whole period at 37 °C in a 95% O_2_/5% CO_2_ incubator. At 1 h before oxygen and glucose deprivation, arginine (100 μL) was administrated.

### Transwell

Neuron were seeded at the basal chamber and microglia seeded in apical chamber Transwell dish were pretreated with arginine and cultured at 37 °C for 24 h. After washing with PBS for 3 times, Oxygen-glucose deprivation insult was applied. At the end of OGD (Oxygen-glucose deprivation) treatment, neuron viability and LDH released were measured.

### Transfection

PCR amplified mouse Hif-1a and LDHA was separately cloned into the pcDNA3.1/hypro (+) vector between *BamH I* and *Not I*, and *Nhe I* and *Xho I*, respectively. pcDNA3.1(+)-Hif-1a–WT (WT Hif-1a) and pcDNA3.1(+)-LDHA–WT (WT LDHA) were generated using the QuikChange Site-Directed Mutagenesis Kit (Stratagene, La Jolla, CA).

### Western blotting analysis

Western blotting was performed as previously described [23]. Briefly, a polyvinylidene difluoride membrane from Millipore was used to incubate the samples with the primary HIF-1α (rabbit, 1:1000, MA1–516, Thermo Fisher, USA), LDHA (mouse, 1:500, sc137244, Santa Cruz, USA) and β-actin (rabbit, 1:2000, bs-0061R, Bioss, China). The primary Antibodies were labeled with corresponding horseradish peroxidase-conjugated secondary antibodies, and protein bands were imaged using SuperSignal West Femto Maximum Sensitivity Substrate (Pierce, Rockford, IL). The EC3 Imaging System (Uplant, UVP) was used to obtain blot images directly from the polyvinylidene difluoride membrane. The Western blot data were quantified using Image-Pro Plus version 6.0.

### RT PCR

Total RNA was extracted from groups of control, sham and treatment by RNeasy Mini Kit (74,106, Qiagen, Germany) according to the manufacturer’s instructions; the first strand of cDNA was synthesized using 5-mg Superscript First Strand Synthesis System for RT-PCR (11,904,018, Invitrogen, USA). PCR was performed on the Opticon 2 Real-Time PCR Detection System (Bio-Rad) using the corresponding primers (Table 1) and FAST SYBR Green PCR Master Mix (4,385,610, Invitrogen, USA). The cycle time value was normalized to the GAPDH level of the same sample. The mRNA expression level was then reported as the fold change compared with the control group.

**Table 1:** Primers of RT-PCR

Gene	Primer
**M1**	
**iNOS**	SENS: 5′-CAAGGAAGGTTGGCATTTGT-3′ REVS: 5′-CCTTTCAGTCCTTTGCAAGC-3′
**TNF-a**	SENS: 5′-ACCACGCTCTTCTGTCTACT-3′ REVS: 5′-GTTTGTGAGTGTGAGGGTCTG-3′
**CD32**	SENS: 5′-AATCCTGCCGTTCCTACTGATC-3′ REVS: 5′-GTGTCACCGTGTCTTCCTTGAG- 3′
**M2**	
**Arg-1**	SENS: 5′-TCACCTGAGCTTTGATGTCG-3′ REVS: 5′-CTGAAAGGAGCCCTGTCTTG-3′
**CD206**	SENS: 5′-CAAGGAAGGTTGGCATTTGT-3′ REVS: 5′-CCTTTCAGTCCTTTGCAAGC-3′
**YM-1**	SENS: 5′-CAGGGTAATGAGTGGGTTGG-3′ REVS: 5′-CACGGCACCTCCTAAATTGT-3’

### Analysis of lactate dehydrogenase release and cell viability

The lactate dehydrogenase (LDH) is a cytoplasmic enzyme retained by viable cells with intact plasma membranes and released from cells with damaged membranes. LDH release was analyzed by a colorimetric CytoTox 96 Cytotoxicity kit (Promega). Cell viability in the neuronal cultures was evaluated by CCK8 assay (BA00208, Bioss, China). The two methods were performed under the manufacturer’s instructions.

### Neurological severity scores

The rats were subjected to a modified neurologic severity score test as reported previously. These tests are a battery of reflex, sensory, motor, and balance tests, and are similar to the contralateral neglect tests in humans. Neurological function was graded based on a scale of 0 to 18 (normal score, 0; maximal deficit score, 18) [24].

### Beam-walk test

The beam-walk test was to measure the complex neuromotor function of animals. The animal was timed as it walked across a (100 × 2 cm) beam. A box for the animal to feel safe was placed at one end of the beam. A loud noise to stimulate the animal to walk toward and into the box. Scoring was based on the time rat spent to go into the box. Higher score reflects more severe neurologic deficit [25].

### Adhesive-removal test

A modified sticky-tape (MST) test was performed to evaluate forelimb function. A sleeve was created using a 3.0 3 1.0-cm piece of yellow paper tape and was subsequently wrapped around the forepaw so that the tape attached to itself and allowed the digits to protrude slightly from the sleeve. The typical response is that the rats vigorously attempt to remove the sleeve by either pulling at the tape with its mouth or brushing the tape with its contralateral paw. The rat was placed in its cage and observed for 30 s. Two timers were started; the first ran without interruption and the second was turned on only while the animal attempted to remove the tape sleeve. The ratio of the left (affected)/right (unaffected) forelimb performance was recorded. The contralateral and ipsilateral limbs were tested separately. The test was repeated three times per test day, and the best two scores of the day were averaged. Lower ratio reflects more severe neurologic deficit [26].

### Statistics

The data and statistical analysis comply with the recommendations on experimental design and analysis. All population data were expressed as mean ± SE. The Student’s t-test or the ANOVA test was used when appropriate. Statistical significance was placed at *P* < 0.05.

## Results

### Arginine reduces infarct volume and improves functional recovery after rat cerebral I/R injury

We tested the effect of arginine in a rat model of cerebral I/R injury, the MCAO. Arginine (1.0 mg/kg) was administrated in contralateral cerebral ventricle at 1 h after MCAO and the infarct volume was measured at 24 h after I/R injury. We found that arginine significantly decreased the infarct volume in animals following I/R injury compared to the vehicle treatment group (Supplement Fig. 1a). In the neurobehavioral tests, the administration of arginine promoted functional recovery of stroke animals at 7 and 14 d after cerebral I/R injury ((Supplement Fig. 1b-d). Together, these results indicate that arginine is neuroprotective in rat cerebral I/R injury.

### Arginine protects against neuronal death via microglia after OGD insult

To investigate how arginine-induced neuroprotection is occurred, we cultured primary neurons with microglia in a Transwell system. The neurons were cultured in the lower plate while the microglia were planted in the upper chamber, which allows the microglial-conditioned media perfuse into the neuronal culture. Before the two cultures were subjected to OGD in the Transwell system, microglia were pretreated with arginine (200 μM) for 1 h. Compared with the neurons in the Transwell system in which microglia were not treated with arginine, neurons in the Transwell system in which microglia treated with arginine show increased viability and decreased LDH release (Supplement Fig. 2a and b). These results suggest that arginine confers neuroprotection in OGD-insulted neurons by regulating co-cultured microglia and its releasing substances.

### Arginine suppresses microglia-mediated inflammatory response after cerebral I/R injury

**Fig. 1:**
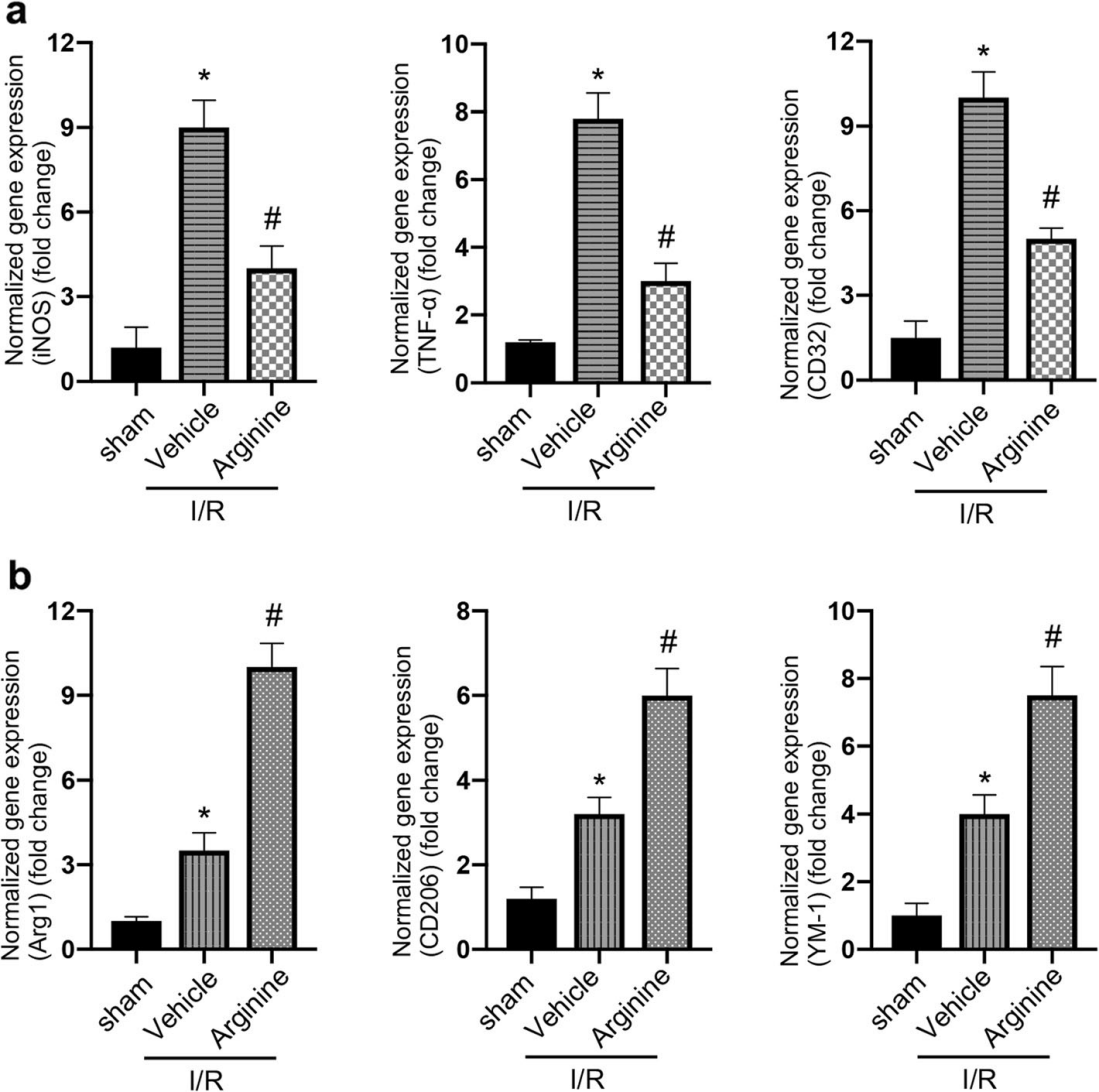
Arginine inhibits inflammatory response after ischemia/reperfusion injury. **a**, **b** RT-PCR analysis shows pro-inflammation is inhibited and anti-inflammation is enhanced in MCAO rats by arginine. RT-PCR measures the expression level of pro-inflammatory markers, iNOS, TNF-α and CD32 (**a**) and anti-inflammatory markers, Arg1, YM-1 and CD206 (**b**) in MCAO rats (*n* = 6 in each group, *p < 0.05 versus sham, # p < 0.05 versus I/R + vehicle, one-way ANOVA test)

As microglia mediate the inflammation process in ischemia stroke [27], we set up to test whether the neuroprotective effect of arginine against neuronal death is mediated by suppressing microglia-mediated inflammatory response in ischemia stroke, we first performed RT-PCR to measure the mRNA expression of both pro-inflammation and anti-inflammation markers in the ischemic rat brain. While administration of arginine increases the levels of pro-inflammation markers, iNOS, TNF-α and CD32, after cerebral I/R injury (Fig. 1a), arginine reduces the expression of anti-inflammation markers, Arg1, YM-1 and CD206 in MCAO animals (Fig. 1b). Thus, we hypothesize that arginine exerts its neuroprotective effect by suppressing microglia-mediated inflammatory reaction after rat cerebral I/R injury.

**Fig. 2:**
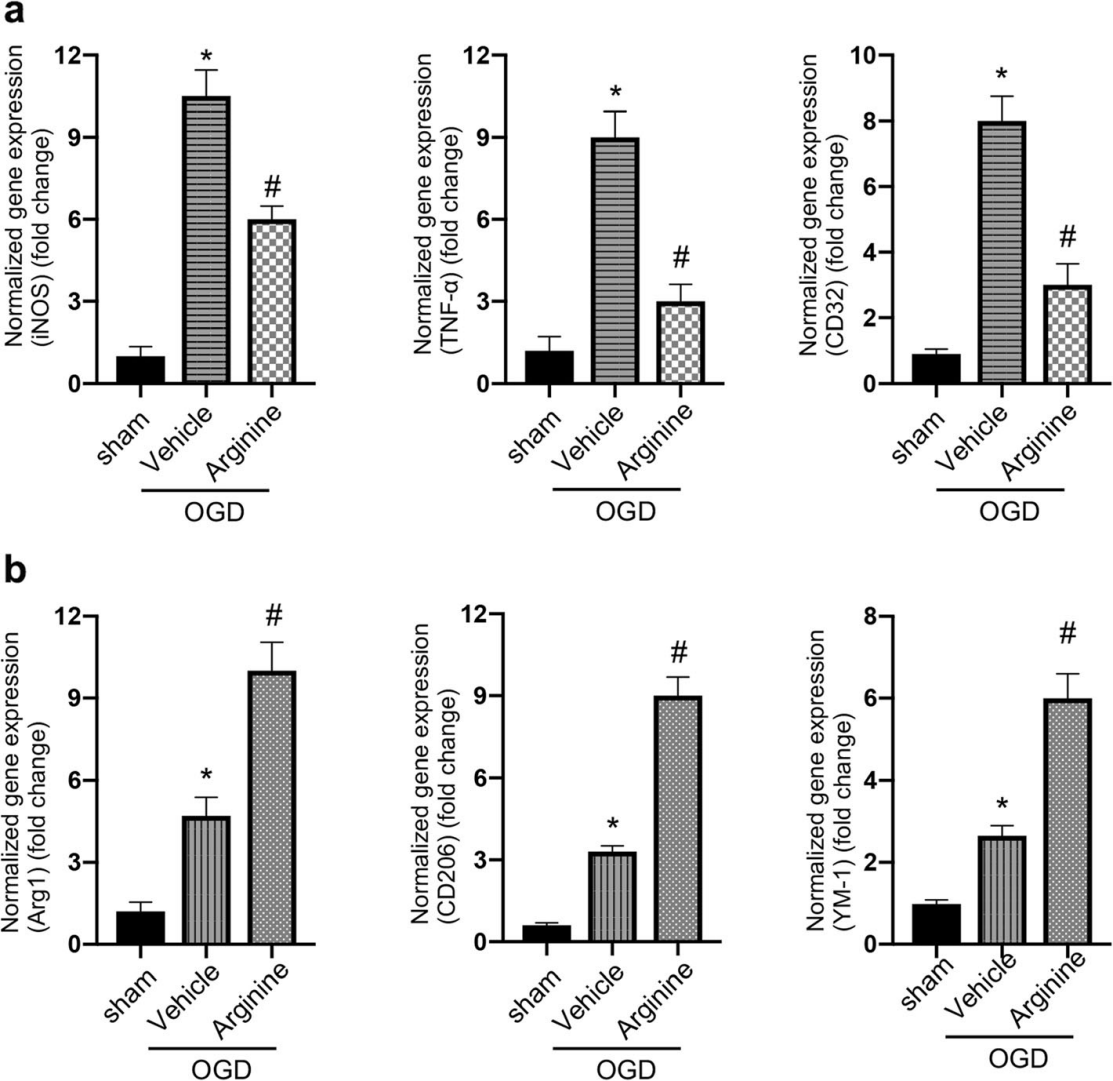
Arginine inhibits inflammation response in primary microglia in vitro after OGD insult. **a**, **b** RT-PCR analysis shows pro-inflammation is decreased and anti-inflammation is enhanced in OGD microglia by arginine. RT-PCR measures the expression level of pro-inflammatory markers, iNOS, TNF-α and CD32 (**a**) and anti-inflammatory markers, Arg1, YM-1 and CD206 (**b**) in OGD microglia (n = 6 in each group, *p < 0.05 versus sham, # p < 0.05 versus I/R + vehicle, one-way ANOVA test)

To verify whether the neuroprotective effect of arginine against neuronal death is mediated through the suppression of the inflammatory response in microglia, we measured the mRNA expression of pro-inflammation markers, iNOS, TNF-α and CD32, in microglia culture after OGD insult. Our results show that arginine administration decreases the pro-inflammation markers in microglia (Fig. 2a). Meanwhile, the anti-inflammation markers, Arg1, YM-1 and CD206 are significantly increased by the treatment of arginine (Fig. 2b). Since microglia is shown to play a critical role in mediating the inflammation process in ischemia stroke [27], our data suggest a possibility that arginine confers neuroprotection through inhibition of microglia-mediated inflammatory response after I/R injury.

### Arginine suppresses HIF-1α/LDHA signaling in microglia after cerebral ischemia injury

**Fig. 3:**
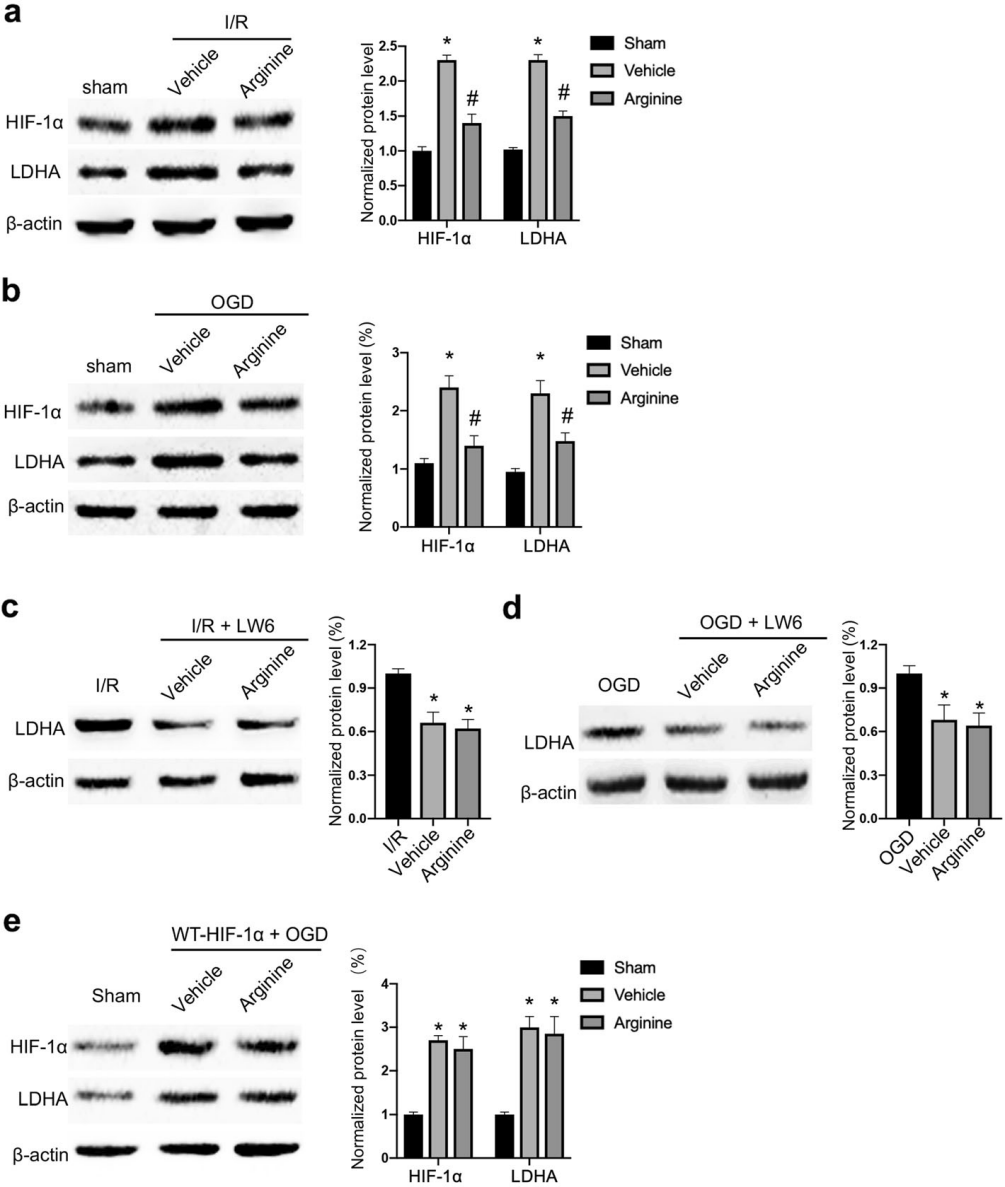
Arginine inhibits HIF-1α to reduce LDHA expression after cerebral I/R injury. **a** Western blotting shows that HIF-1α and LDHA are raised in MCAO rats and the increase is attenuated by arginine administration (n = 6 in each group, *p < 0.05 versus sham, # p < 0.05 versus vehicle, two-way ANOVA test). **b** Western blotting shows that HIF-1α and LDHA are raised in in OGD microglia and the increase is attenuated by arginine administration (n = 6 in each group, *p < 0.05 versus sham, # p < 0.05 versus vehicle, two-way ANOVA test). **c** Western blotting shows that administration of the HIF-1α inhibitor, LW6 decreases the protein level of LDHA in MCAO rats and occludes the effect of arginine (n = 6 in each group, *p < 0.05 versus vehicle, one-way ANOVA test). **d** Western blotting shows that administration of LW6 decreases the level of LDHA in microglia and occludes the effect of arginine (n = 6 in each group, *p < 0.05 versus OGD, one-way ANOVA test). **e** Western blotting shows that transfection of WT-HIF-1α abolishes arginine-induced LDHA reduction in OGD BV-2 cells (n = 6 in each group, *p < 0.05 versus OGD, one-way ANOVA test)

To explore the molecular mechanism by which arginine alleviates the inflammatory response, we performed western blotting to measure the protein level of HIF-1α and LDHA. The level of HIF-1α and LDHA are increased at 24 h after rat cerebral I/R injury (Fig. 3a). However, arginine administration downregulates HIF-1α and LDHA at 24 h after rat cerebral I/R injury (Fig. 3a). In primary microglia culture, the protein levels of HIF-1α and LDHA are increased at 24 h after OGD insult. Whereas arginine administration prevents the increase of HIF-1α and LDHA at 24 h after OGD insult (Fig. 3b).

HIF-1α/LDHA signaling is involved in mediating inflammatory response [17, 20]. To determine whether arginine suppresses microglia-mediated inflammatory response through downregulating HIF-1α/LDHA signaling after cerebral I/R injury, we tested the effect of LW6, a HIF-1α inhibitor, on LDHA level (Supplement Fig. 3a and b)*.* We show that treatment of LW6 (20 mg/kg) 1 h before MCAO reduces the level of LDHA at 24 h after MCAO (Fig. 3c). Administration of LW6 (4.4 μM) 1 h before OGD also reduces the level of LDHA at 24 h after OGD insult in primary microglia culture (Fig. 3d). In addition, the level of LDHA expression is remarkably elevated after transfecting HIF-1α cDNA in BV-2 cells, a mouse microglia cell line (Supplement Fig. 3c and Fig. 3e). These results indicate that HIF-1α positively regulates LDHA in microglia after cerebral I/R injury.

To validate whether HIF-1α/LDHA is the downstream target of arginine under ischemia stroke condition, we tested the effect of LW6 in MCAO rats. LW6 administration reduces the level of LDHA in the ischemic brain, and the effect of arginine on LDHA is blocked by LW6 (Fig. 3c). LW6 administration also occludes arginine-induced decline of LDHA after OGD in cultured primary microglia (Fig. 3d). Further, in BV-2 cell line, overexpression of HIF-1α abolishes the effect of arginine on decreasing the level of LDHA after OGD insult (Fig. 3e). Collectively, these results lead us to conclude that arginine may suppress HIF-1α/LDHA signaling in microglia after rat cerebral I/R injury.

### Arginine inhibits inflammatory response in microglia via suppressing HIF-1α/LDHA signaling after cerebral I/R injury

**Fig. 4:**
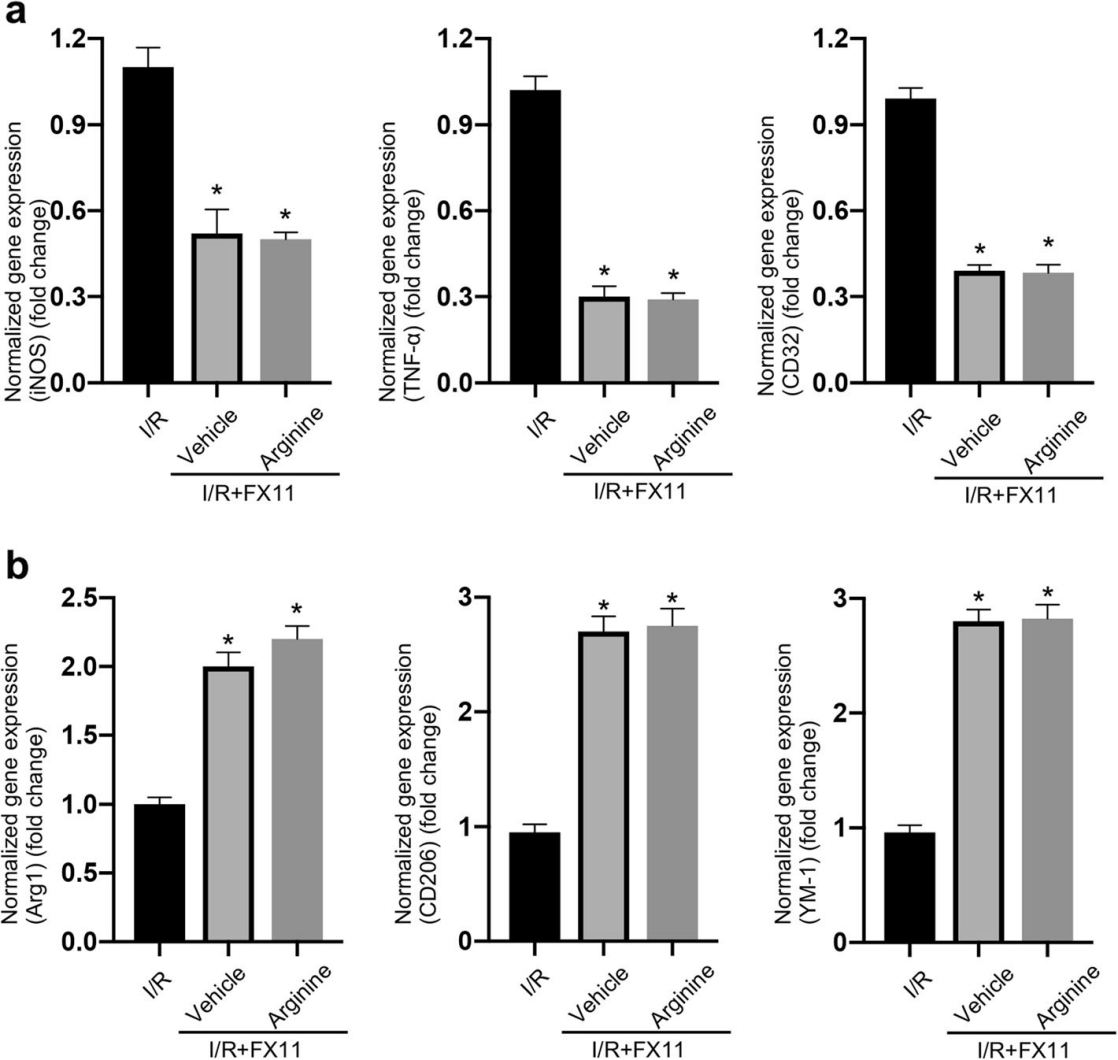
Arginine inhibits MCAO-induced inflammatory response by the suppression of LDHA. **a** RT-PCR shows that the increased pro-inflammatory response in MCAO rats is inhibited by FX11. FX11 occludes the pro-inflammation inhibition of arginine (n = 6 in each group, *p < 0.05 versus I/R, one-way ANOVA test). **b** RT-PCR shows that anti-inflammation is enhanced by FX11 in MCAO rats. FX11 occludes anti-inflammation upregulation by arginine (n = 6 in each group, *p < 0.05 versus I/R, one-way ANOVA test)

**Fig. 5:**
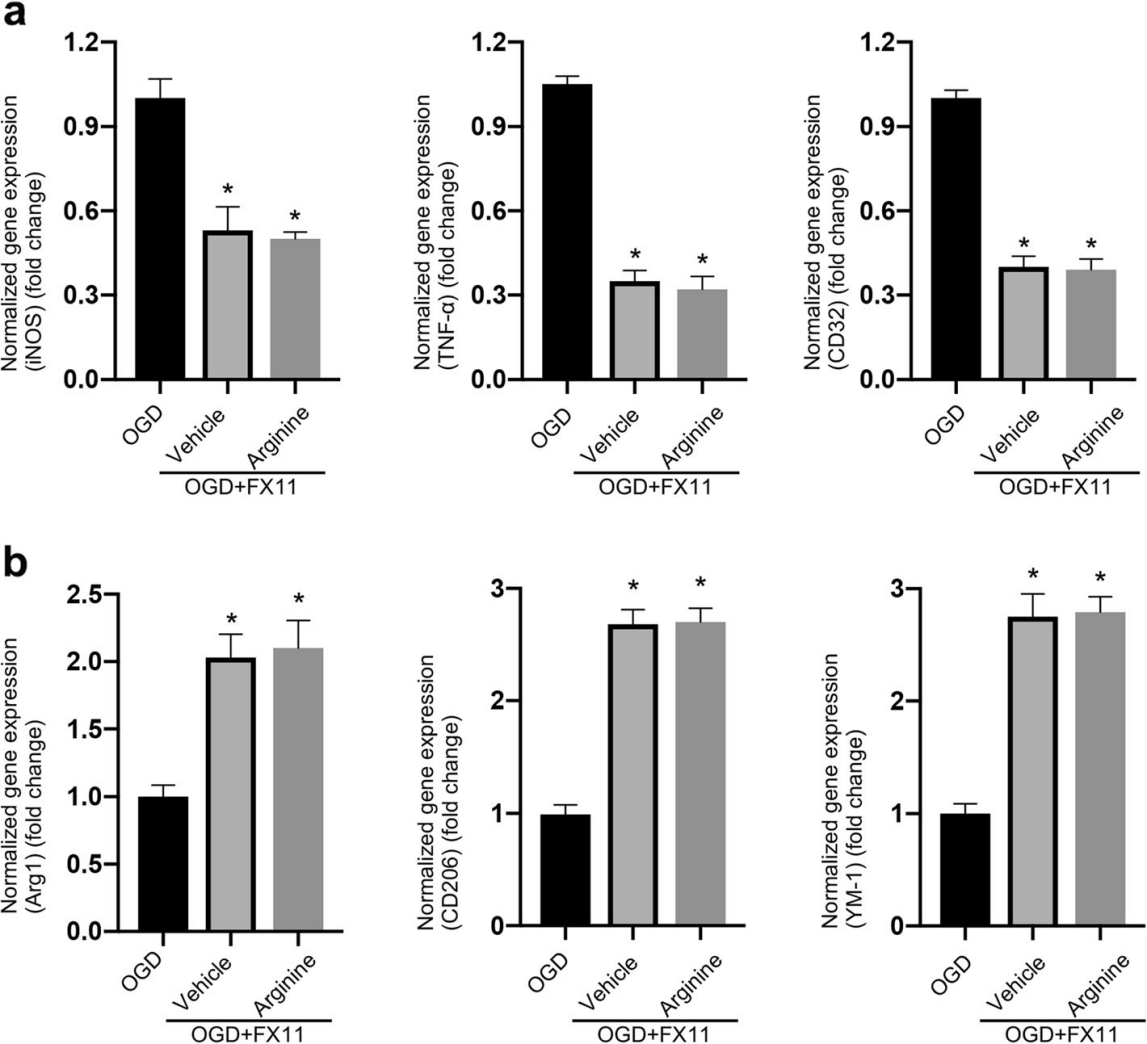
Arginine inhibits OGD-induced inflammatory response by suppression of LDHA in microglia. **a** RT-PCR shows that pro-inflammatory response in OGD microglia is inhibited by FX11. FX11 occludes the pro-inflammation inhibition of arginine (n = 6 in each group, *p < 0.05 versus OGD, one-way ANOVA test). **b** RT-PCR shows that anti-inflammation in OGD microglia is enhanced by FX11. FX11 occludes the anti-inflammation upregulation by arginine (n = 6 in each group, *p < 0.05 versus OGD, one-way ANOVA test)

In order to determine whether arginine attenuates inflammatory response by inhibiting HIF-1α/LDHA in ischemia stroke, we tested the markers of pro-inflammation and anti-inflammation in MCAO rats treated with FX11 (an LDHA inhibitor) and arginine. FX11 (2.2 mg/kg) was treated 1 h before MCAO and followed by arginine administration. We found that FX11 suppress the increasing of inflammation response at 24 h after rat cerebral I/R injury (Fig. 4a and b). However, FX11 occludes the effects of arginine on both the decrease of pro-inflammation markers and increase of anti-inflammation markers (Fig. 4a and b). In primary cultured microglia, FX11 (10 μM) was added to the cultures at 1 h before OGD. FX11 inhibits the increase of inflammation response at 24 h after OGD insult (Fig. 5a and b). Consistent with the in vivo results, FX11 occludes the effect of arginine on the down-regulation of pro-inflammatory markers and the up-regulation of anti-inflammatory markers (Fig. 5a and b).

We then transfected LDHA cDNA in BV-2 cells (Supplement Fig. 4a). Overexpression of LDHA blocks the effect of arginine on LDHA (Supplement Fig. 4b). Consistently, the inhibitory effect of arginine on pro-inflammation markers and the elevation of anti-inflammation markers by arginine are abolished by LDHA overexpression in BV-2 cell line (Supplement Fig. 5a and b). These results suggest that arginine suppresses inflammatory response by inhibiting microglia-mediated HIF-1α/LDHA pathway.

### Arginine confers neuroprotection by suppressing HIF-1α/LDHA-mediated inflammation response in microglia after cerebral I/R injury

**Fig. 6:**
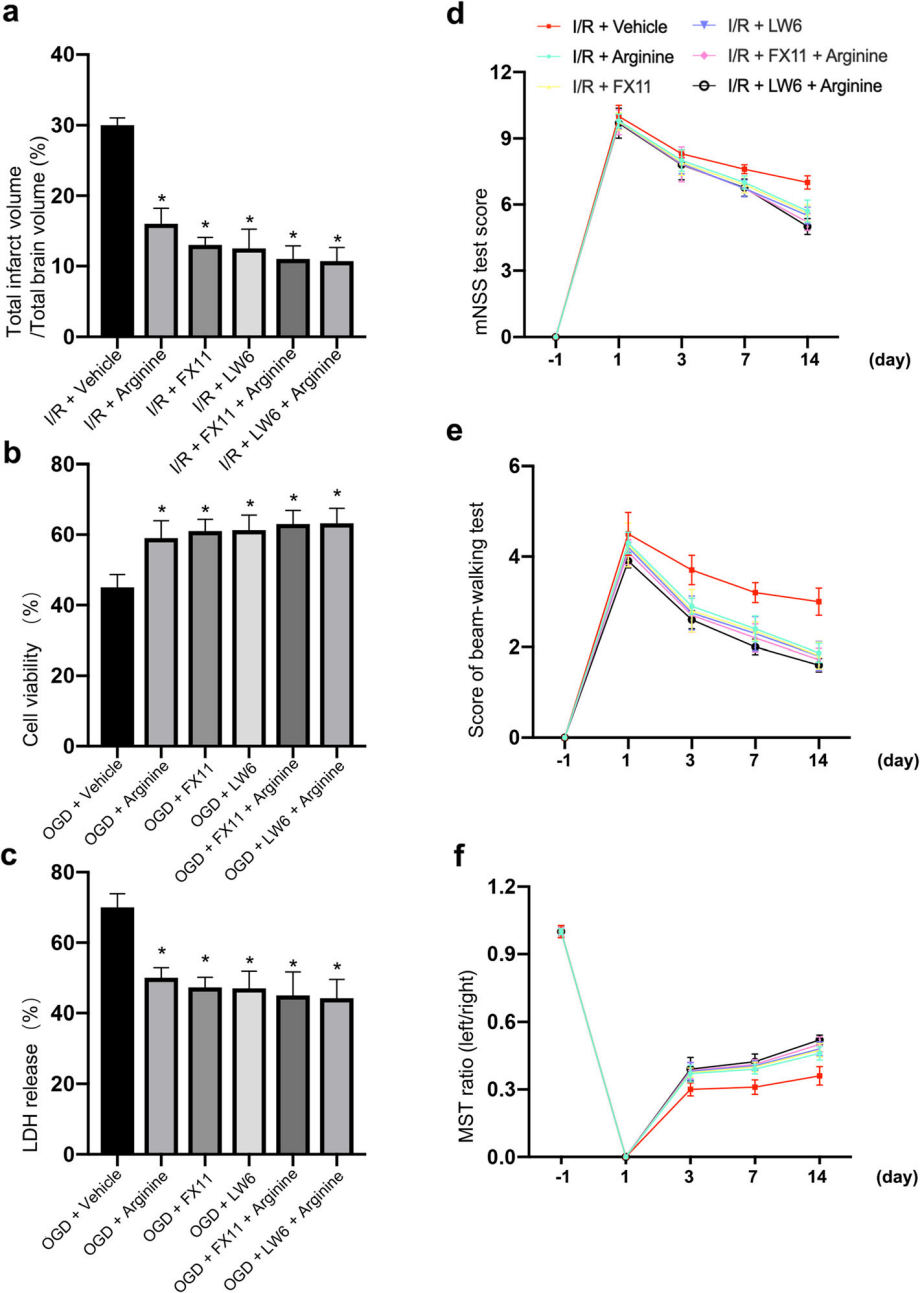
Arginine prevents ischemic neuronal death by inhibiting inflammation response via suppression of HIF-1α/LDHA pathway. **a** TTC test shows arginine is neuroprotective, and no significant difference in infarct volume among arginine group, FX11 group, LW6 group, FX11 + arginine group and LW6 + arginine group in MCAO rats (n = 6 in each group, *p < 0.05 versus vehicle, one-way ANOVA test). **b**, **c** Cell viability test **(b)** and LDH release **(c)** show that administration of arginine in OGD microglia attenuates co-cultured neuron death, and no significance among arginine group, FX11 group, LW6 group, FX11 + arginine group and LW6 + arginine group (n = 6 in each group, *p < 0.05 versus vehicle, one-way ANOVA test). **d**, **e**, **f** mNSS test **(d)**, beam-walking test **(e)**, and MST test **(f)** show that there is no significant difference of functional recovery among arginine group, FX11 group, LW6 group, FX11 + arginine group and LW6 + arginine group (n = 6 in each group, *p < 0.05 versus Vehicle, two-way ANOVA test)

Our data thus far prove that arginine inhibits inflammatory response by suppressing HIF-1α/LDHA signaling in microglia after ischemia stroke. To test whether the arginine/HIF-1α/LDHA pathway is neuroprotective after cerebral ischemia injury, we carried out TTC test. We found that the LW6 and FX11 occludes the arginine-induced infarct volume decrease in MCAO rats, respectively (Fig. 6a). This indicates that the neuroprotective effect of arginine is carried out by inhibition of HIF-1α/LDHA pathway.

To test whether the neuroprotective effect of arginine is by suppressing microglia-mediated HIF-1α/LDHA pathway, we co-cultured microglia and neuron in the Transwell plate as we previously described. After OGD insult, cell viability test and LDH release show that the neuroprotective effect of arginine is occluded by administration of LW6 and FX11 respectively as well (Fig. 6b and c). Results demonstrates that HIF-α/LDHA inhibition in microglia after ischemia stroke is neuroprotective. In addition, we performed neurobehavioral tests to testify whether the arginine-induced improved functional recovery is executed by HIF-1α/LDHA suppression. Data showed that FX11 and LW6 could occlude the promoted behavioral recovery of arginine respectively (Fig. 6d-f). In conclusion, arginine plays the neuroprotection role by attenuating inflammatory response after ischemia stroke via inhibiting HIF-1α/LDHA signaling.

## Discussion

During the second stage of ischemia stroke, events such as oxidant stress, blood-brain barrier breakdown and inflammatory response, cause secondary injury [5]. The investigation of post-ischemia neuroinflammation, due to its complexity, is still limited [15, 28]. Arginine has been reported to exert neuroprotective effect [12]. Nevertheless, the underlying mechanisms are largely unknown. In this study, we demonstrate that the neuroprotective effect of arginine is exerted by attenuating inflammatory response in microglia and reveal the mechanism by which the suppression of inflammatory response is mediated through inhibition of HIF-1α/LDHA pathway in ischemia stroke.

Microglia is the primary neural cell in charge of immunity. While confronted with ischemia stroke, microglia are activated. Although inflammation mediated by microglia could be neuroprotective at the first stage, the continuous inflammatory response causes neuronal injury [29]. There are two phenotypes of activated microglia, one phenotype exerts pro-inflammatory effect while the other one is anti-inflammatory. These two states of microglia are defined as M1 and M2. Pro-inflammation microglia release pro-inflammatory molecules, such as iNOS, TNF-a, IL-1β, CD16/32 and IL-1β [30, 31]. For the anti-inflammation phenotype of microglia, they release anti-inflammatory molecules, including Arg1, Ym1, CD206 and IL-4 [32]. These molecules are used as markers to represent the anti-inflammatory state and pro-inflammatory state of microglia, respectively. Pro-inflammation response promotes neuronal apoptosis. On the contrary, the anti-inflammation is neuroprotective and benefits the recovery after injury [7, 33]. Thus, by attenuating pro-inflammation and enhancing anti-inflammation, neuronal death could be alleviated through the process of inhibiting inflammatory response, such as phagocytosis [34, 35]. In this study, we measured the level of pro-inflammation and anti-inflammation markers and find that the inflammatory response is inhibited by arginine administration.

Arginine is a non-essential amino acid and has been reported to inhibit active oxidative stress, acute inflammatory response, and hence alleviate cell injury. Arginine also promotes the production of NO (nitric oxides) to rescue vascular damage [36–38]. Regulation of arginine metabolism is reported to be neuroprotective by administration of melatonin after MCAO [39]. Arginine metabolism is in the charge of Arg1 and iNOS, which respectively represents anti-inflammation effect and pro-inflammation effect [40, 41]. However, how arginine exerts its neuroprotective effect and the molecular mechanism underlying arginine-induced inhibition of inflammatory response are not well studied in ischemia stroke. In this study, we probe into the inflammatory pathway after administration of arginine in cerebral ischemia injury. By measuring the level of pro-inflammation and anti-inflammation markers, we found that arginine suppresses the inflammatory response in MCAO rat brain and cultured primary microglia suffered from OGD. Whether arginine has direct neuroprotective effect on neurons subjected to OGD insult requires further studies. Our data suggest that administration of arginine could be a potential neuroprotection strategy by inhibiting the inflammatory microglial response after ischemia stroke.

Our results reveal that the molecular mechanism of arginine-mediated neuroprotection is through the suppression of HIF-1α/LDHA signaling pathway and the subsequent inhibition of inflammatory response. It has been reported that regulation of glycolysis alters the inflammatory response in microglia [42]. HIF-1α and LDHA are glycolysis-related proteins [42]. HIF-1α alters transcription processes in hypoxia condition and positively regulates pro-inflammatory response [43, 44]. In ischemia stroke, the level of HIF-1α level is increased [21, 45]. Although our results suggest that HIF-1α inhibition exerts neuroprotective in our experimental conditions, other study indicates that neuron-specific inactivation of HIF-1α increases brain injury in a mouse model of transient focal cerebral ischemia [46]. The different effect of HIF-1α on microglia and neuron, the different signaling pathways that coupled to HIF-1α and other possible reasons may underlie the discrepancy. Further investigations are required to answer the questions.

As a transcription factor, HIF-1α positively mediates LDHA [47]. Under conditions such as hypoxia and multiple sclerosis, the expression of LDHA is increased, and inhibition of LDHA suppresses the inflammation reaction [20, 48]. However, the LDHA-induced inflammatory response has not yet been studied in ischemia stroke. We found that the HIF-1α/LDHA signaling are enhanced in response to ischemia stroke and that HIF-1α positively regulates LDHA in ischemia stroke. Our results further demonstrate that HIF-1α/LDHA is the downstream of arginine. LW6 is a HIF-1α inhibitor and FX11 is a LDHA inhibitor [49]. FX11 is reported to suppress macrophage and prohibit inflammatory cytokines by LDHA inhibition [50]. By measuring the mRNA level of pro-inflammation and anti-inflammation marker, our data showed that the treatment of LW6 and FX11 occluded the inflammation suppression effect induced by arginine in MCAO rats and cultured primary microglia subjected to OGD. Consequently, arginine inhibits inflammatory response in microglia by inhibiting HIF-1α/LDHA signaling after cerebral ischemia insult.

Arginine is the part of urea cycle, and is reported to promote cell proliferation. While lacking arginine, glycolysis is inhibited and cell proliferation could be blocked [14]. This indicates that there may be synergistic effect between urea cycle and glycolysis mediated by arginine.

In summary, our study demonstrates that arginine confers neuroprotection by downregulating HIF-1α/LDHA signaling in microglia and thus inhibit the inflammatory response after ischemia stroke. Our results suggest that arginine treatment is potential neuroprotective strategy by suppressing microglial inflammatory response via attenuating the HIF-1α/LDHA signaling in ischemia stroke.

## Supplementary information

**Additional file 1: Supplement Fig. 1.** Arginine is neuroprotective after rat cerebralischemia/reperfusion injury. **Supplement Fig. 2**. Arginine administration to microglia co-cultured with neuron in Transwell system is neuroprotective in OGD insult. **Supplement Fig. 3.** Regulation of LDHA by HIF-1α. **Supplement Fig. 4**. Regulation of LDHA by arginine after transfection of LDHA and OGD. **Supplement Fig. 5**. Arginine-mediated inhibition of inflammatory response is blocked by transfection of LDHA in OGD BV-2 cell line.

## Data Availability

Please contact author for data requests.
