# Supplementary material for: Arginine is neuroprotective through suppressing HIF-1α/LDHA-mediated inflammatory response after cerebral ischemia/reperfusion injury
Source: Mol Brain. 2020 Apr 22;13:63. doi: 10.1186/s13041-020-00601-9 (PMC7175589; doi:10.1186/s13041-020-00601-9)
Supplement: Supplementary file 1 — Additional file 1: Supplement Fig. 1. Arginine is neuroprotective after rat cerebralischemia/reperfusion injury. Supplement Fig. 2. Arginine administration to microglia co-cultured with neuron in Transwell system is neuroprotective in OGD insult. Supplement Fig. 3. Regulation of LDHA by HIF-1α. Supplement Fig. 4. Regulation of LDHA by arginine after transfection of LDHA and OGD. Supplement Fig. 5. Arginine-mediated inhibition of inflammatory response is blocked by transfection of LDHA in OGD BV-2 cell line. [file 13041_2020_601_MOESM1_ESM.pdf]

Arginine is neuroprotective through suppressing HIF-1α/LDHA-mediated inflammatory response after cerebral ischemia/reperfusion injury

Song-Feng Chen[1*], Meng-Xian Pan[1*], Jun-Chun Tang[1], Jing Cheng[2], Dan Zhao[3], Ya Zhang[1], Hua-Bao Liao[1], Rui Liu[1], Yang Zhuang[1], Zhi-Feng Zhang[3], Juan Chen[4], Rui-Xue Lei[1], Shi-Fang Li[5], Huan-Ting Li[5], Ze-Fen Wang[1], Qi Wan[5]

[1] Department of Physiology, School of Basic Medical Sciences, Wuhan University School of Medicine, 185 Donghu Street, Wuhan 430071, China
[2] Department of Neurosurgery, Renmin Hospital of Wuhan University, 99 Zhang Zhidong Rd, Wuhan 430060, China
[3] Department of Physiology, School of Basic Medical Sciences, Hubei University of Medicine, 30 South Renmin Road, Shiyan, Hubei, 442000 China
[4] Department of Neurology, the Central Hospital of Wuhan, Tongji Medical College, Huazhong University of Science & Technology, 26 Shengli Street, Wuhan 430013, China
[5] Institute of Neuroregeneration & Neurorehabilitation, Department of Neurosurgery & Pathophysiology, Qingdao University, 308 Ningxia Street, Qingdao 266071, China

*These authors contributed equally to this work.

Correspondence:
Qi Wan, Institute of Neuroregeneration & Neurorehabilitation, Qingdao University, 308 Ningxia Street, Qingdao 266071, China
Email: qwanwh@hotmail.com
Ze-Fen Wang, Department of Physiology, School of Basic Medical Sciences, Wuhan University School of Medicine, 185 Donghu Street, Wuhan 430071, China
Email: 964434321@qq.com

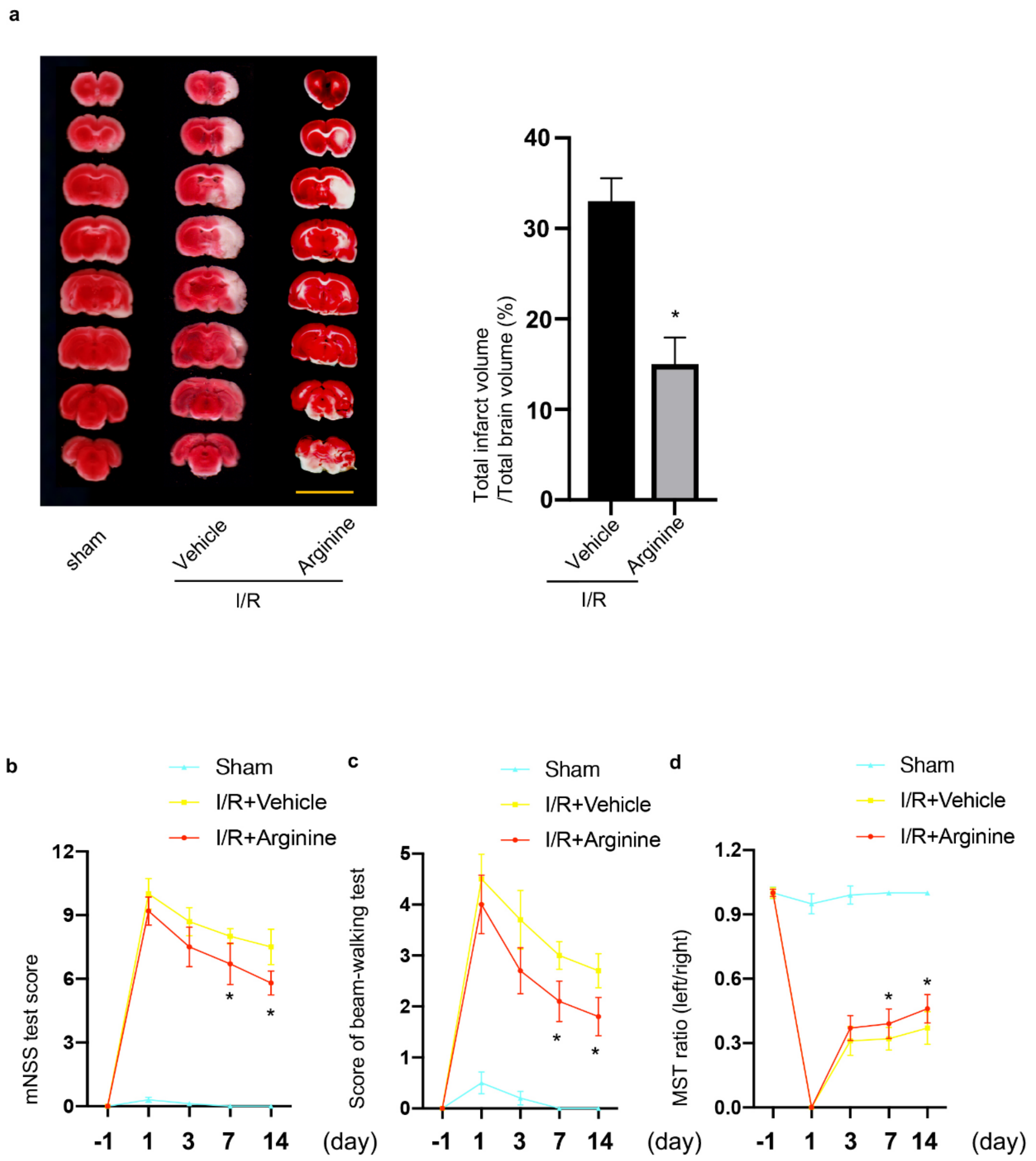

Supplement Fig 1. Arginine is neuroprotective after rat cerebral ischemia/reperfusion injury. (**a**) TTC staining (left) shows the infarct region after 24 h following I/R insult and quantification analysis (right) of the infarct volume shows that arginine administration reduces I/R-induced infarct volume (n = 6 in each group, *p < 0.05 versus the I/R + vehicle, Student's t-test). (Scale bar, 5 mm). (**b**) Arginine treated rats have lower scores in the mNSS test on days 7 and 14 after I/R injury compared to rats in the I/R + vehicle group. (n = 6 in each group, *p < 0.05 versus I/R + vehicle, two-way ANOVA test). (**c**) Arginine treated rats have lower scores in the beam-walking test on days 7 and 14 after I/R injury compared to rats in the I/R + vehicle group. (n = 6 in each group, *p < 0.05 versus the I/R + vehicle, two-way ANOVA test). (**d**) Arginine treated rats have higher a ratio in the MST test at days 7 and 14 after I/R injury compared to rats in the I/R + vehicle group (n = 6 in each group, *p < 0.05 versus the I/R + vehicle, two-way ANOVA test).

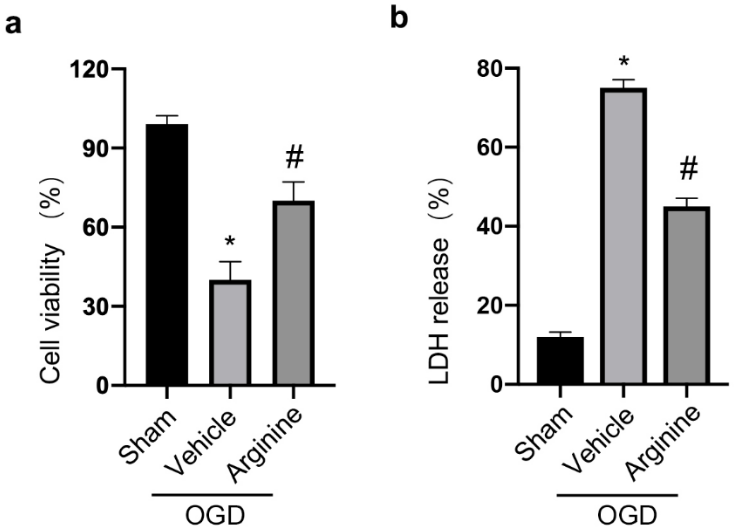

Supplement Fig 2. Arginine administration to microglia co-cultured with neuron in Transwell system is neuroprotective in OGD insult. (a, b) Cell viability test **(a)** and LDH release test **(b)** show that administration of arginine to microglia reduces neuron death after OGD insult (n = 6 in each group, *p < 0.05 versus Sham, #p < 0.05 versus Vehicle, one-way ANOVA test).

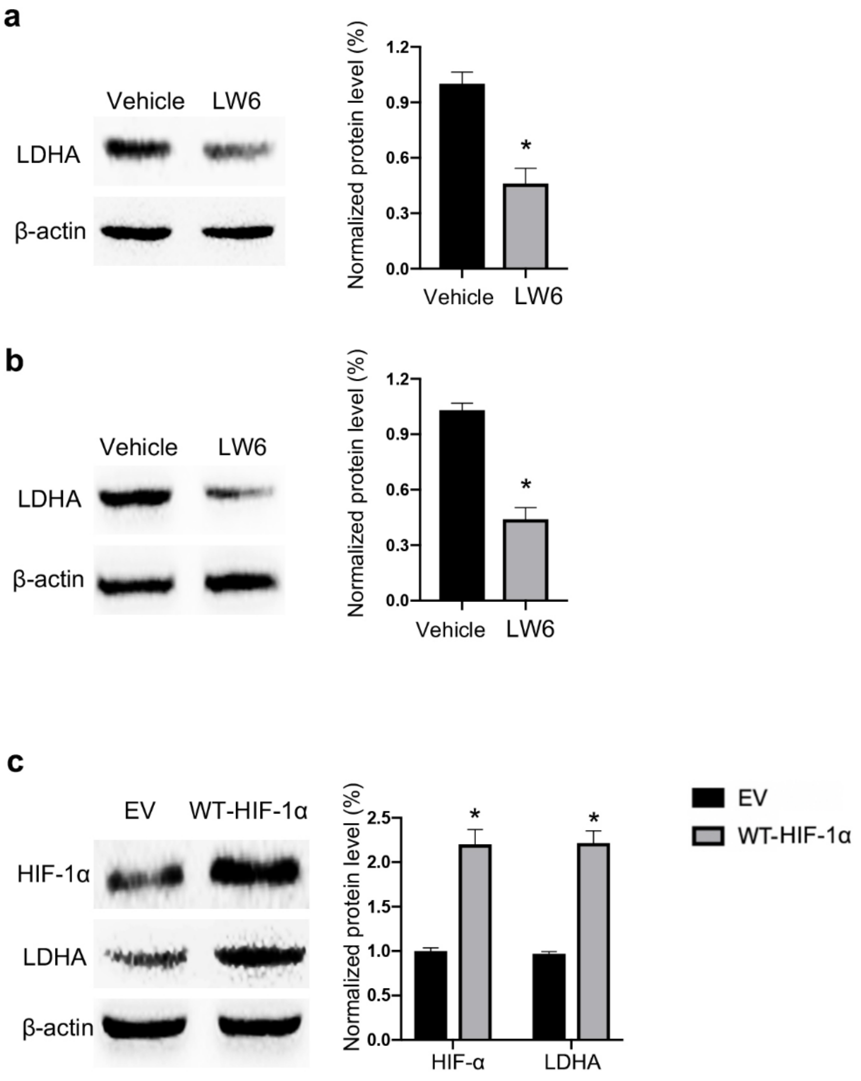

Supplement Fig 3. Regulation of LDHA by HIF-1α. (a, b) Western blotting shows that LW6 inhibits LDHA protein level in rats **(a)** and cultured primary microglia **(b)** (n = 6 in each group, *$p < 0.05$ versus vehicle, Student's t-test). **(c)** Western blotting shows transfection of WT-HIF-1α increases LDHA protein level in BV-2 cell line (n = 6 in each group, *$p < 0.05$ versus EV, two-way ANOVA test).

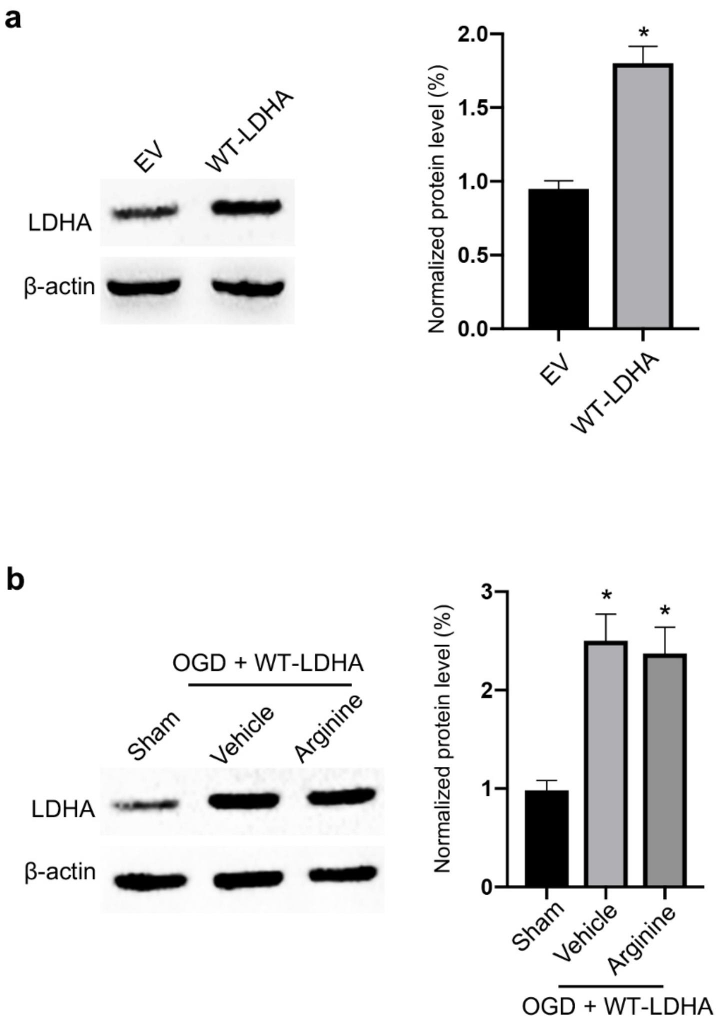

Supplement Fig 4. Regulation of LDHA by arginine after transfection of LDHA and OGD. (a) Western blotting shows transfection of LDHA upregulates LDHA expression in BV-2 cell line (n = 6 in each group, *p < 0.05 versus EV, Student's t-test). **(b)** Western blotting shows LDHA overexpression maintains the expression level of LDHA after OGD insult in BV-2 cell line administrated with arginine (n = 6 in each group, *p < 0.05 versus OGD, one-way ANOVA test).

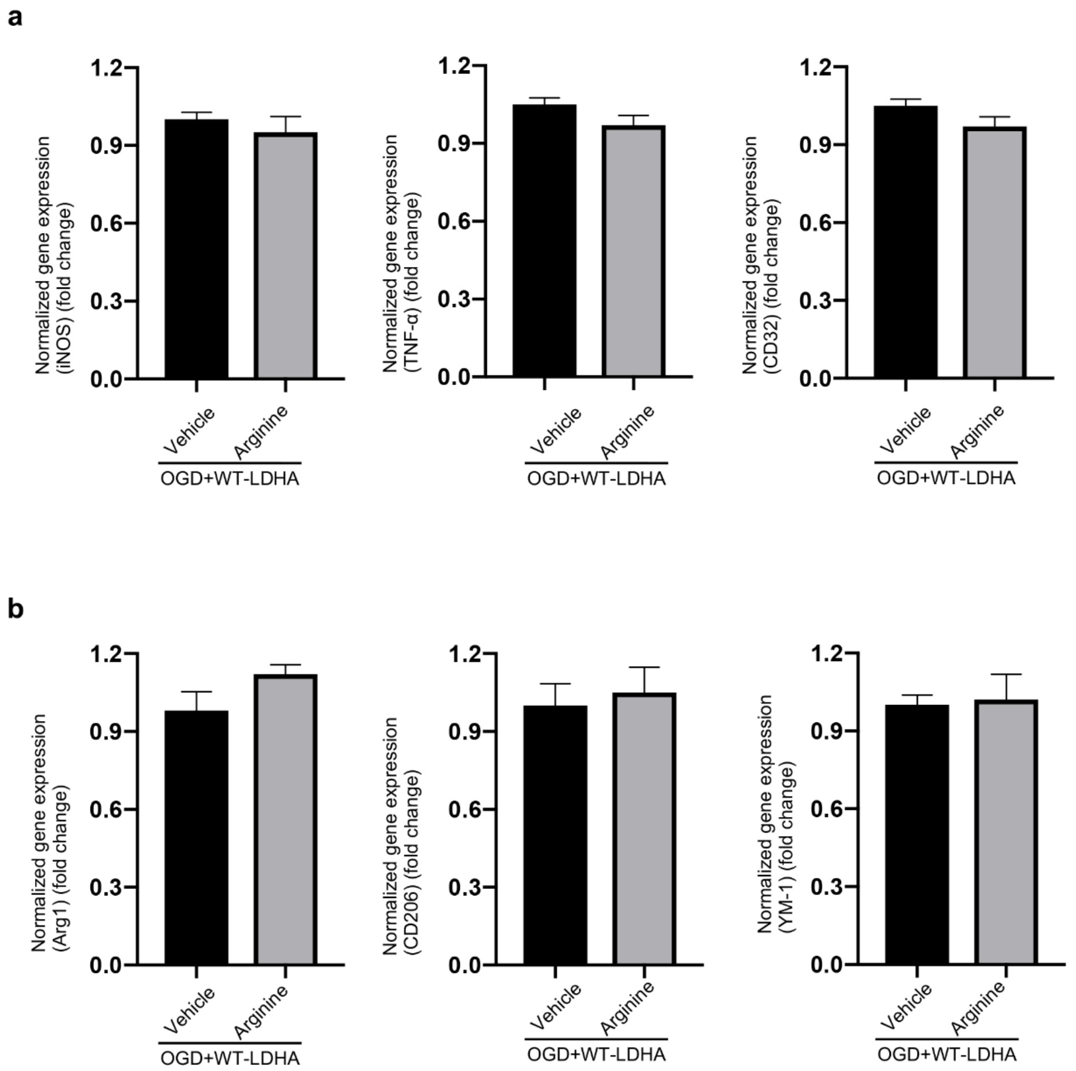

Supplement Fig 5. Arginine-mediated inhibition of inflammatory response is blocked by transfection of LDHA in OGD BV-2 cell line. (a, b) Pro-inflammatory markers **(a)** and anti-inflammatory markers **(b)** in FX11 group showed no significance from FX11 + arginine group (n = 6 in each group, Student's t-test).
